# How Did You Get There? The Value of Segues for Illustrating Your Logic

**DOI:** 10.5334/pme.2647

**Published:** 2026-05-26

**Authors:** Kevin W. Eva

**Affiliations:** 1 Centre for Health Education Scholarship, University of British Columbia, Canada

## Abstract

In the writer’s craft section we offer simple tips to improve your writing in one of three areas: Energy, Clarity and Persuasiveness. Each entry focuses on a key writing feature or strategy, illustrates how it commonly goes wrong, teaches the grammatical underpinnings necessary to understand it and offers suggestions to wield it effectively.

I want you to think about a bridge. No, not that one. I presume, at least, that you envisioned one of the world’s engineering marvels that stand majestically as art – the Tower Bridge, the Golden Gate, Sydney Harbour. Instead, I want you to think about one of the bridges that span a creek or valley in your part of the world that is so well-blended into the road or boardwalk on either side that it is easy to miss. While invisible to most, these structures serve an essential purpose, creating a path that is contiguous and cohesive by virtue of not drawing attention.

In writing, music and conversation, such a bridge is what we call a segue. Pronounced “seg-way,” it is the smooth transition, without interruption, from one topic, song, or activity to another. Did you see it at the start of this paragraph? I could have offered a first (topic) sentence that simply read “In writing, music and conversation, segues are smooth transitions, without interruption…”. By adding a simple allusion – “such a bridge” – I attempted to maintain connection to the metaphor established in the preceding paragraph (backward signaling [1]) while simultaneously transitioning to where the text is going (a consideration of segues more generally).

Why should we care? There it is again – four words that make it appropriate to start a new paragraph (because they clearly indicate a new topic), yet create a segue by connecting what is to come to what was just read. Drawing attention to transitions in this way makes them anything but seamless, so I’ll stop doing that in favour of answering the question posed (after parenthetically noting this sentence to be an example of forward signaling, foreshadowing with a paragraph’s final (wrap) sentence what is to come in the next paragraph [1]).

Segues are critically responsible for demonstrating the logic underlying our arguments (telling the story of our study in Lingard and Watling’s terminology [2]). Their elegance derives from how easy they are to miss given that segues rarely contribute new content to the prose [3]. Derived from Italian for “it follows,” segues instead demonstrate to the reader why the author believes it to be sensible that point b follows from point a. It is a matter of “showing your work,” as a math teacher might say, strengthening arguments by offering linkages within and between them. Segues enhance fluency by reducing the cognitive load created when readers need to decipher the author’s logic by themselves. They are, in other words, the difference between text that reads like a set of puzzle pieces strewn on the page for the reader to put together and text that enables the reader to easily see the connections necessary to appreciate the full picture. Put simply, segues are a fundamental determinant of coherence.

The topic of coherence in our writing is so significant that at least two previous articles in the Writer’s Craft series have been dedicated to it. Lingard wrote about intra-paragraph coherence, outlining how the structure of a paragraph can be used strategically to encapsulate a unit of thought [4]. Co-authoring with Foo, she has also effectively drawn attention to inter-paragraph coherence, offering techniques to connect those thought units (such as the forward and backward signaling noted above). Coherence, however, has been such a prominent part of the conversations I find myself having when giving feedback about writing that it seemed worth expanding upon both articles by demonstrating how coherence can be more deeply woven into our writing. To do so, I offer an array of segue types followed by tips for using them effectively.

## Segue Types

To this point, I have offered examples of segues based on where they are presented in the text – we can end a paragraph with words that foreshadow what is to come (forward signaling) or start a paragraph with a reminder of what just transpired (backward signaling). Such transitions, however, are important throughout our writing, enabling connections to be drawn between clauses, between sentences, between paragraphs, and between sections [5]. They can be single words (“However,” “Although”), phrases (“That said,” “If true,” “It follows that”), or complete sentences (“To fully appreciate how stereotypes shape attitudes, it is necessary to examine…”) [3].

Beyond where or what though, it can help to also think about segues in terms of the function you aim to achieve [6]. Ideally, you have chosen to sequence your ideas (at both the sentence and paragraph level) purposefully. Was that to enable a **comparison** (“Similarly,” “Likewise”)? To establish a **contrast** (“On the other hand,” “Conversely”)? To provide **elaboration** (“At the same time,” “More specifically”)? To add **information** (“Furthermore,” “Beyond that”)? To draw an **implication** (“As a result,” “Hence”)? To **clarify** (“Put simply,” “Said another way”)? To offer **examples** (“As evidenced by,” “To illustrate”)? Or, to **orient** readers to the organization of subsequent text (“Before doing that,” “Initially”)?

These small bits of text can seem like niceties, especially when word limits are rigid. And, it is true that they are not always necessary. Yet, consider what they enable in the following text, paraphrased from above:

“To this point, I have offered examples of segues based on where they are presented in the text. Segues, however, are important throughout our writing. As such, it may help to also think in terms of their function.”

“To this point” and “however” both serve contrasting functions, forewarning the reader that something is about to change. The word “also” serves a clarifying function – remove it and the text no longer tells the reader that I am trying to complement the forward vs backward taxonomy previously mentioned rather than contradicting it. Furthermore, the phrase “As such” explains that I think the third sentence to be a direct implication of the second. Remove these words and you are left with the following:

“I have offered examples of segues based on where they are presented in the text. Segues are important throughout our writing. It may help to think in terms of their function.”

In addition to being rather stilted, that text is less clear regarding whether the second sentence builds on the first, is a pivot point in the argument, or amounts to a tangential aside. Furthermore, this example illustrates that while “connecting words” (segues) most often start a sentence, text that smooths transitions can be inserted anywhere that it fits, which is helpful to avoid the tedium induced by reading prose that is rigidly and repetitively structured.

## Segue Tips

1. Think about the forest AND the trees while revising

Most advice on revising manuscripts focuses on what to do in response to peer reviewers [e.g., 7]. One of the most impactful things you can do to improve your writing, however, is undertake many rounds of revision before any reviewer has the opportunity to comment. In doing so, don’t just read and re-read your manuscript in search of typographic or grammatical errors. Rather, make dedicated passes at revision with an eye on cohesion and unity by looking specifically at your segues. The strength of an argument can change dramatically upon re-sequencing sentences or full paragraphs in a way that enables more compelling links (i.e., a more logical flow) to be made between them through segues. Other times, smoothness is lost because authors miss the need to update their segues upon deciding to re-sequence text. Always focusing on the details (e.g., spelling, word choice) can be a distraction that blinds us to larger structural issues. Carefully contemplating the connections you draw with segues (again, within and between sentences as much as between paragraphs) offers a direct test of whether you have effectively drawn attention to what you intend to emphasise.

2. Be concise

The longer the text, the lengthier your segues are likely to be. In dissertations and non-fiction books, for example, introductory chapters commonly segue to the rest of the book with paragraph-length descriptions of what will follow (“In chapter 2, I will … Following that …”). In shorter prose such as scientific articles, the purpose statement, hypothesis or research question that commonly transitions the reader from the Introduction into the Methods is a one-sentence example of the same thing. Even more extreme, most of the examples of segues highlighted in this manuscript have been 1 to 3 words in length, providing evidence that you need not add a lot of words to enable clearer connection between them.

3. Vary your approach

To ensure you have room for segues that add to your text’s fluency, take care to avoid redundancy and repetitiveness. There is no value to be drawn, for example, from ending one paragraph with “As such, segues are a fundamental determinant of coherence” only to redundantly start the next one by stating “Illustrative of why segues are a fundamental determinant of coherence…” In terms of repetitiveness, we all have specific phrases that we tend to revisit, but doing so can cause readers’ eyes to blur if we are not conscious about finding a variety of ways to address the functions listed in the preceding section. English contains more synonyms than any language in the world [8]; I would venture a guess that contributes to it also being most flexible in sentence and paragraph structure. Use that to your advantage when choosing your segues to keep readers engaged. To build your repertoire for the sake of avoiding overuse, more examples of segue phrases are offered in Box 1. That list is inevitably non-comprehensive and illustrates only “a” (rather than “the”) common function served because what each word or phrase does depends on how it is used. For example, I have listed “Although” both as having a contrastive function (e.g., Although that is an important consideration, we argue that …) and an elaboration function (e.g., Although the list in Box 1 is impressive, we can add to it by…). “Although” could also be used for clarification and comparison along with other functions. Say it with me … “English is flexible.”

**Box 1 An inevitably non-comprehensive list of segue options with which you can experiment, categorized by a common function they serve**

**Table:** 

**Addition –** Demonstrations that one piece of text is an expansion of another		
Also	And	Besides
Beyond that	Coupled with	Finally
First, Second, Third…	Furthermore	In addition
Indeed	Moreover	Taken together
**Clarification –** Demonstrations that two pieces of text express the same idea in alternate ways		
In other words	Namely	One could say
Put differently	Put simply	Said another way
That is	To clarify	What that means is
**Comparison –** Demonstrations that two pieces of text are related to one another		
Also	Analogous to this	By comparison
By the same token	Consistent with	Correspondingly
Equally important	In the same vein	In the same way
Less vital	Likewise	Much like
Resembling	Similarly	Too
**Contrast –** Demonstrations that two pieces of text oppose one another		
Alternatively	Although	Conversely
Despite that	However	Instead
Nonetheless	On the contrary	On the other hand
Rather	Still	That said
To this point	While	Yet
**Elaboration –** Demonstrations that one piece of text expands upon another		
Although	At the same time	Even more extreme
In doing so	In fact	In particular
In terms of	More specifically/generally	Taken at face value
What is more	Whereby	Widening on that point
**Exemplification –** Demonstrations that one piece of text reinforces a more general statement		
As case in point	As evidenced by	For example
For instance	Illustrative of that	Namely
These	To illustrate	To wit
**Implication –** Demonstrations that one piece of text is a logical outcome of another		
As a result	As such	Because
Consequently	Ergo	For that reason
Given that observation	Hence	If true
It follows that	Since	That suggests
Then	Therefore	Thus
**Orientation –** Demonstrations of how connections will be made between sections of text		
Before doing that we will	Beginning with	In doing so
In this chapter	Initially	To do so
To ensure that	To that end	Ultimately we will

4. Align your segue’s function with the text you wish to connect

A segue is useful for establishing coherence only when it is used accurately. Returning to the paraphrased text from above, consider what happens otherwise:

“To this point, I have offered examples of segues based on where they are presented in the text. Segues, therefore, are important throughout our writing.

“Therefore” is an implication segue. That makes its use here illogical, confusing matters rather than adding clarity – the importance of segues is not determined by the fact I previously offered examples; nor that I offered them in a particular way. As a result, “therefore” is a red herring, bewildering the reader by undermining the logic of the connection. That not only makes the text jarring, but risks miscommunication (and irritation) as the reader has to guess at what the author meant. Is it that “segues, however, are important throughout our writing” or that “segues being categorizable based on where they occur in a text makes them important”? In my impression, implication segues and contrast segues are particularly prevalent in cases of mis-use.

5. Don’t force it

Some readers will have noticed that I made oddly little attempt to segue between tips given that this section is about using segues well. That was a deliberate effort to demonstrate the value of subheadings. Take away “Segue Tips” from above and the transition between this section and the paragraph that precedes it would be unsettling. I could have smoothed that, instead of using a subheading, by inserting “With that in mind, let’s consider strategies for using segues effectively.” By inserting a subheading, however, I aimed to give the reader a moment to pause consolidate the argument that had been put forward in the preceding section while creating more digestible chunks of information. If the content does not allow a natural and logical transition to be illustrated (or if inserting one runs the risk of overwhelming readers’ working memory), that may be a symptom of needing to re-think your argument. Is an intervening idea required to smooth the connection? Is an intervening sentence breaking the smooth transition from one idea to another (and, hence, better deleted)? Can you achieve better transitions by re-ordering your sentences or paragraphs? If none of these things are true, it might be an indication that you should excuse yourself from including a direct segue by inserting a subheading. Do so sparingly, however, and note that there could still be value in indirectly stating connections like when I signaled what was to come at the end of the introductory paragraphs through a sentence-long orientation segue:

“To do so, I offer an array of segue types followed by tips for using them effectively.”

## Conclusion

Authors should not be surprised that readers do not follow their logic when the logic is not explicitly laid out. Segues between clauses, sentences, paragraphs, and sections are a critical means of showing how each idea expressed relates to those around it. Unfortunately, their existence, let alone their value, is often overlooked by novice writers because the elegance and value of segues is often defined by how seamlessly they create bridges that carry the reader from one claim to the next. Authors, thus, are advised to deliberately attend to what type of segue is required to enable such transitions, altering the way they express themselves if they cannot make a segue work effectively, and varying the specific segues used to enable cohesion and fluency.
